# The LSD1-Type Zinc Finger Motifs of *Pisum sativa* LSD1 Are a Novel Nuclear Localization Signal and Interact with Importin Alpha

**DOI:** 10.1371/journal.pone.0022131

**Published:** 2011-07-19

**Authors:** Shanping He, Kuowei Huang, Xu Zhang, Xiangchun Yu, Ping Huang, Chengcai An

**Affiliations:** The State Key Laboratory of Protein and Plant Gene Research, College of Life Sciences, Peking University, Beijing, China; University of South Florida, United States of America

## Abstract

**Background:**

Genetic studies of the *Arabidopsis* mutant *lsd1* highlight the important role of LSD1 in the negative regulation of plant programmed cell death (PCD). *Arabidopsis thaliana* LSD1 (AtLSD1) contains three LSD1-type zinc finger motifs, which are involved in the protein–protein interaction.

**Methodology/Principal Findings:**

To further understand the function of LSD1, we have analyzed cellular localization and functional localization domains of *Pisum sativa* LSD1 (PsLSD1), which is a homolog of AtLSD1. Subcellular localization analysis of green fluorescent protein (GFP)-tagged PsLSD1 indicates that PsLSD1 is localized in the nucleus. Using a series of GFP-tagged PsLSD1 deletion mutants, we found that the three LSD1-type zinc finger motifs of PsLSD1 alone can target GFP to the nucleus, whereas deletion of the three zinc finger motifs or any individual zinc finger motif causes PsLSD1 to lose its nuclear localization, indicating that the three zinc finger motifs are necessary and sufficient for its nuclear localization. Moreover, site-directed mutagenesis analysis of GFP-tagged PsLSD1 indicates that tertiary structure and basic amino acids of each zinc finger motif are necessary for PsLSD1 nuclear localization. In addition, yeast two-hybrid, pull-down, and BiFC assays demonstrate that the three zinc finger motifs of PsLSD1 directly bind to importin α *in vitro* and *in vivo*.

**Conclusions/Significance:**

Our data demonstrate that the LSD1-type zinc finger motifs of PsLSD1 are a novel nuclear localization signal and directly bind to importin α, and suggest that the nuclear import of LSD1 may rely on the interaction between its zinc finger motifs and importin α. Moreover, the nuclear localization of PsLSD1 suggests that LSD1 may function as a transcription regulator involved in negatively regulating PCD.

## Introduction

Genetic analyses of *Arabidopsis lsd1* mutant have indicated that LSD1 is an important negative regulator of plant programmed cell death (PCD) [1], [2]. In addition, genetic analyses of the *lsd1* mutant have shown that LSD1 is involved in regulating salicylic acid (SA) induction of copper zinc superoxide dismutase, and negatively regulating reactive oxygen species (ROS) and stress-induced ethylene levels [2], [3], [4], [5], [6], [7]. *Arabidopsis thaliana* LSD1 (AtLSD1) contains three LSD1-type zinc finger motifs (InterPro accession number: IPR005735), which are defined by CxxCRxxLMYxxGASxVxCxxC [8] and are involved in interacting with other proteins [9], [10], [11].

Nuclear import is essential for nuclear proteins, such as transcription factors, to execute their function in the nucleus. The classical nuclear import pathway involves the interaction between a classical nuclear localization signal (NLS) and a heterodimeric import receptor [12]. In general, the classical NLSs fall into two categories: monopartite and bipartite NLS [12]. The monopartite NLS is characterized by a short stretch of basic amino acids, such as PKKKRKV in the SV40 large T antigen protein [13]. The bipartite NLS is first identified in nucleoplasmin and characterized by two interdependent sets of basic amino acids, which are separated by an approximately ten amino acid linker [14]. However, NLSs without basic amino acid clusters have been reported in recent years [15], [16], [17], [18], [19]. The heterodimeric import receptor, consisting of importin α and β, is involved in the nuclear import of proteins containing a classical NLS [12]. Importin α directly binds to the classical NLS of cargos and importin β, forming a trimeric complex [12]. Importin β mediates the transportation of the trimeric complex into the nucleus through interacting with the nuclear pore complex [12].

Since the subcellular localization may provide valuable information on the mode of action of LSD1, we set out to further study its cellular localization and to determine its localization domains by using our cloned *Pisum sativa LSD1* (*PsLSD1*) cDNA. In this study, we identify PsLSD1 as a nuclear protein and further identify the three LSD1-type zinc finger motifs of PsLSD1 as a novel NLS. Moreover, the zinc finger motifs of PsLSD1 directly bind to importin α *in vitro*. Thus, our results provide important information for the nuclear localization mechanism of LSD1 and suggest that LSD1 may serve as a transcription regulator implicated in the negative regulation of PCD.

## Results

### PsLSD1 is involved in negatively regulating PCD

Previously, we cloned a reduced glutathione (GSH)-induced pea cDNA, which encodes a LITAF domain protein, and named it *Pisum sativa GSH-induced LITAF domain protein* (*PsGILP*; GenBank accession number: AAY40471.1). To identify PsGILP-interacting proteins, we used yeast two-hybrid screen to clone a pea cDNA (GenBank accession number: HQ006097). This cDNA encodes a putative protein homologous to AtLSD1 (with 72% identity), and thus was designated as *PsLSD1* (*Pisum sativa LSD1*). The predicted PsLSD1 has 176 amino acid residues and contains three LSD1-type zinc finger motifs (Figure S1).

**Figure 1:**
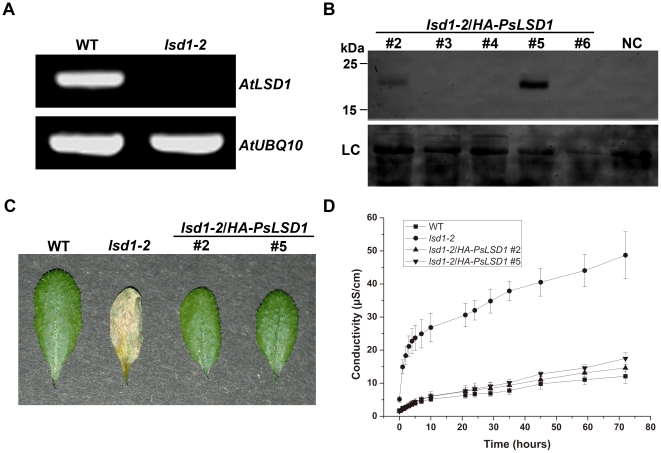
PsLSD1 is involved in negatively regulating PCD. (A) RT-PCR analysis of the expression of *AtLSD1* in the *lsd1-2* mutant. *AtUBQ10* was used as an internal control. (B) Western blot analysis of transgenic plant *lsd1-2*/*HA-PsLSD1* lines using an anti-HA monoclonal antibody. Wild-type plant was used as negative control. Various transgenic lines are numbered; NC and LC represent negative control and loading control, respectively. (C) Lesion phenotypes of various plant lines after SA treatment. Leaves were photographed at 5 days post-SA spray. Each leaf shown is a representative of about 30 leaves in three independent experiments. (D) Ion leakage profiles of various plant lines after SA treatment. At 3 days post-SA spray, conductivity of leaf discs from various plant lines was measured at the indicated time. SD indicates four independent data points. The experiment was performed three times with similar results.

To investigate the function of PsLSD1, we identified an *Arabidopsis* T-DNA insertion line of *AtLSD1* (SALK_042687, designated as *lsd1-2*) (Figure 1A), and obtained two independent transgenic lines over-expressing *PsLSD1* in the homozygous *lsd1-2* mutant (Figure 1B). We sprayed wild-type (WT), *lsd1-2*, and the transgenic lines with SA, which can induce PCD in *lsd1-2*, but not in WT [9]. By 5 days post-SA spray, *lsd1-2* leaves were collapsed and completely dried, indicating significant cell death. In contrast, WT and the transgenic lines were healthy and green (Figure 1C). We quantified cell death by measuring cellular ion leakage, which correlates with plant cell death [20]. As shown in Figure 1D, *lsd1-2* displayed a significant increase in conductivity, whereas the transgenic lines were basically the same as WT and exhibited only a very slight increase in conductivity. Thus, over-expression of *PsLSD1* could rescue SA-induced PCD of *lsd1-2*, indicating that PsLSD1 is involved in negatively regulating PCD.

### PsLSD1 is localized in the nucleus

**Figure 2:**
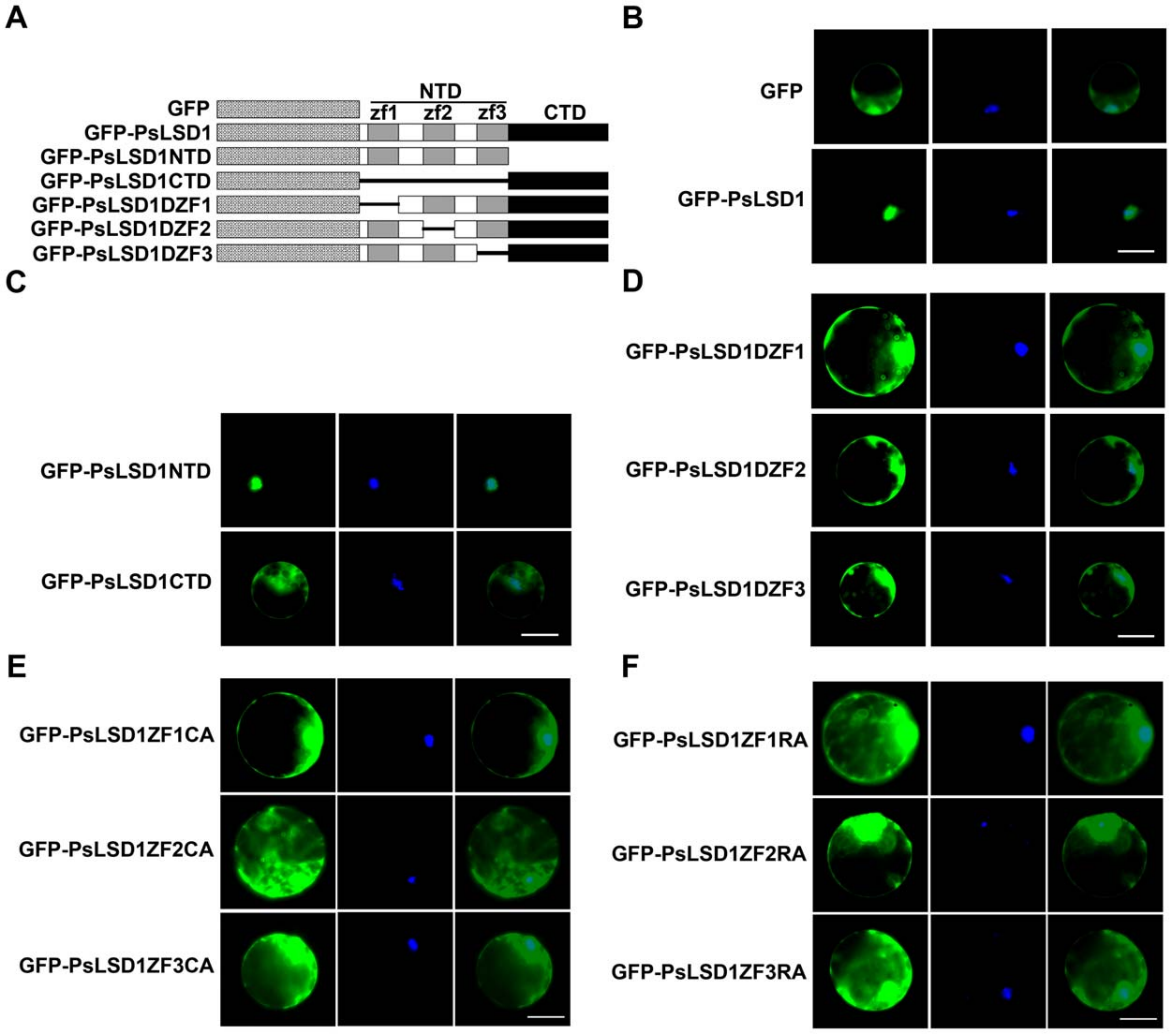
The NLS of PsLSD1 is located in the three LSD1-type zinc finger motifs. (A) Schematic diagram of GFP-tagged PsLSD1 and its deletion mutants. zf1, zf2, and zf3 indicate the first, second, and third zinc finger motif, respectively. NTD and CTD represent the N- and C-terminal domain, respectively. (B) PsLSD1 is localized in the nucleus. (C) The N-terminal domain is sufficient and necessary for PsLSD1 nuclear localization. (D) All the three zinc finger motifs are necessary for PsLSD1 nuclear localization. (E) Tertiary structure of each zinc finger motif is essential for PsLSD1 nuclear localization. ZF1CA, ZF2CA, and ZF3CA indicate the mutation of the second cysteine to alanine within the first, second, and third zinc finger motif, respectively. (F) Basic residues of each zinc finger motif are essential for PsLSD1 nuclear localization. ZF1RA, ZF2RA, and ZF3RA indicate the mutation of the first arginine to alanine within the first, second, and third zinc finger motif, respectively. *Arabidopsis* mesophyll protoplasts were transfected with the indicated constructs, and samples were stained with DAPI to indicate positions of nuclei. Fluorescent images were taken at 12–16 h after transfection. Each protoplast shown is a representative of at least twenty protoplasts in three independent experiments. Epifluorescence (left), DAPI (middle), and merged (right) images are shown. Scale bar is 20 µm.

To determine subcellular localization of PsLSD1, we constructed a fusion of the green fluorescent protein (GFP) gene and *PsLSD1* driven by the CaMV 35S promoter (Figure 2A), and transfected *Arabidopsis* mesophyll protoplasts with the resulting construct. As shown in Figure 2B, GFP alone was distributed throughout the cytoplasm and the nucleus, whereas GFP-PsLSD1 was exclusively localized in the nucleus. This result indicates that PsLSD1 is localized in the nucleus.

### The NLS of PsLSD1 lies within the three zinc finger motifs

No classical NLS was found in the amino acid sequence of PsLSD1. To identify the NLS of PsLSD1, its two domains were separately fused to GFP: the N-terminal domain (NTD, aa 1–105) containing three LSD1-type zinc finger motifs, and the C-terminal domain (CTD, aa 106–176) (Figure 2A). As shown in Figure 2C, GFP-PsLSD1NTD was found predominantly in the nucleus, whereas GFP-PsLSD1CTD was found in the cytoplasm and the nucleus. This result indicates that the N-terminal domain is sufficient and necessary for PsLSD1 nuclear localization.

Since the N-terminal domain of PsLSD1 contains three zinc finger motifs, we next asked which zinc finger motifs are necessary for PsLSD1 nuclear localization. To address this question, we generated three constructs expressing GFP-tagged deletion mutants of PsLSD1 under the control of the CaMV 35S promoter: GFP-PsLSD1ΔZF1 in which aa 1 to 28 were deleted, GFP-PsLSD1ΔZF2 in which aa 46 to 67 were deleted, and GFP-PsLSD1ΔZF3 in which aa 84 to 105 were deleted (Figure 2A). Like GFP, GFP-PsLSD1ΔZF1, GFP-PsLSD1ΔZF2, and GFP-PsLSD1ΔZF3 were distributed in the cytoplasm and nucleus (Figure 2D), indicating that all the three zinc finger motifs are essential for PsLSD1 nuclear localization. Taken together, these results suggest that the three zinc finger motifs are the NLS of PsLSD1.

### Tertiary structure of zinc finger motifs is essential for PsLSD1 nuclear localization

The four zinc-chelating cysteine residues are involved in binding to zinc ion, and thus are essential for tertiary structure of LSD1-type zinc finger motif. To determine whether zinc finger structure is essential for PsLSD1 nuclear localization, we generated three constructs in which the second zinc-chelating cysteine within each zinc finger is mutated to alanine to disrupt zinc finger structure, and analyzed their subcellular localization. As shown in Figure 2E, GFP-PsLSD1ZF1CA, GFP-PsLSD1ZF2CA, and GFP-PsLSD1ZF3CA were distributed in the cytoplasm and nucleus. This result indicates that the tertiary structure of each zinc finger motif is essential for PsLSD1 nuclear localization.

### Basic residues of zinc finger motifs are essential for PsLSD1 nuclear localization

The primary amino acid sequence of PsLSD1 zinc finger motifs contains only seven basic residues: two, three, and two basic residues are present in the first, second, and third zinc fingers, respectively (Figures S1). To examine the role of basic residues in PsLSD1 nuclear localization, we generated three constructs in which the first arginine within each zinc finger is mutated to alanine, and analyzed their subcellular localization. As shown in Figure 2F, GFP-PsLSD1ZF1RA, GFP-PsLSD1ZF2RA and GFP-PsLSD1ZF3RA were distributed in the cytoplasm and nucleus. This result indicates that basic residues within the zinc finger motifs are essential for PsLSD1 nuclear localization.

### The NLS of PsLSD1 binds to importin α

**Figure 3:**
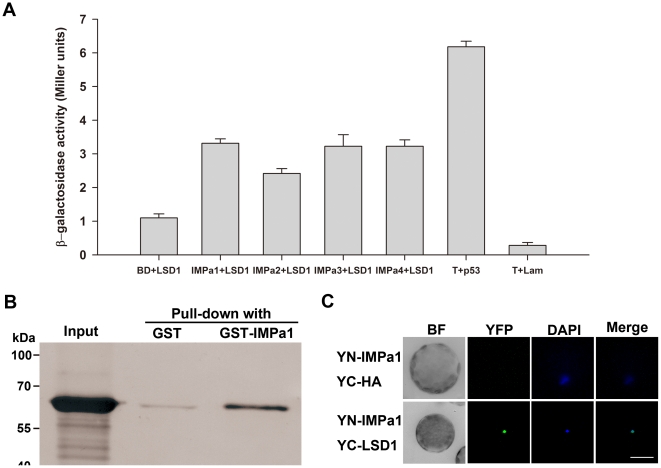
The NLS of PsLSD1 is involved in interacting with importin α. (A) PsLSD1 interacts with AtIMPα1, AtIMPα2, AtIMPα3, and AtIMPα1 in yeast. pGBK-AtIMPα1, pGBK-AtIMPα2, pGBK-AtIMPα3, pGBK-AtIMPα4, and pGBKT7 were co-transformed with pGAD-PsLSD1 into yeast AH109 respectively, and β-galactosidase activity of the resulting clones was measured. “T+p53” and “T+Lam” are positive and negative controls for the yeast two-hybrid assay, respectively. (B) PsLSD1 directly binds to AtIMPα1 *in vitro*. Purified MBP-PsLSD1 was incubated with GST or GST-AtIMPα1 bound to glutathione particles. Pulled-down proteins and “Input” sample (purified MBP-PsLSD1) were detected by Western blot using an anti-MBP polyclonal antibody. (C) PsLSD1 interacts with AtIMPα1 *in vivo*. YN-AtIMPa1 was co-transfected with YC and YC-PsLSD1 into *Arabidopsis* mesophyll protoplasts, respectively, and samples were stained with DAPI to indicate positions of nuclei. Fluorescent images were taken at 12–16 h after transfection. Each protoplast shown is a representative of at least twenty protoplasts in two independent experiments. BF indicates Bright Field, and scale bar is 20 µm.

To elucidate how the NLS of PsLSD1 is involved in the nuclear import of PsLSD1, we analyzed the possible interaction between the NLS of PsLSD1 and nuclear import protein importin α, which recognizes the classical NLSs. The *Arabidopsis* genome contains nine genes encoding importin α isoforms [21]. We initially analyzed the interactions between four of these importin α isoforms (AtIMPα1, AtIMPα2, AtIMPα3, and AtIMPα4) and PsLSD1. Yeast two-hybrid assay showed that all these four importin α proteins associated with PsLSD1 (Figure 3A). To confirm the interaction between PsLSD1 and importin α, we performed *in vitro* GST pull-down assay. Very little MBP-PsLSD1 protein was pulled-down by GST-bound beads, whereas significantly more MBP-PsLSD1 protein was pulled-down by GST-AtIMPα1-bound beads (Figure 3B), indicating that PsLSD1 binds to AtIMPα1 *in vitro*.

To further verify the interaction between PsLSD1 and AtIMPα1, we performed bimolecular fluorescence complementation (BiFC) assay in *Arabidopsis* protoplasts. As shown in Figure 3C, no fluorescence signal was observed in protoplasts co-expressing YN-AtIMPα1 and YC-HA (upper panel), whereas the fluorescence signal was detected in the nucleus of protoplasts co-expressing YN-AtIMPα1 and YC-PsLSD1 (bottom panel). This result indicates that PsLSD1 associates with AtIMPα1 *in vivo*. Taken together, these data demonstrate that PsLSD1 associates with importin α.

**Figure 4:**
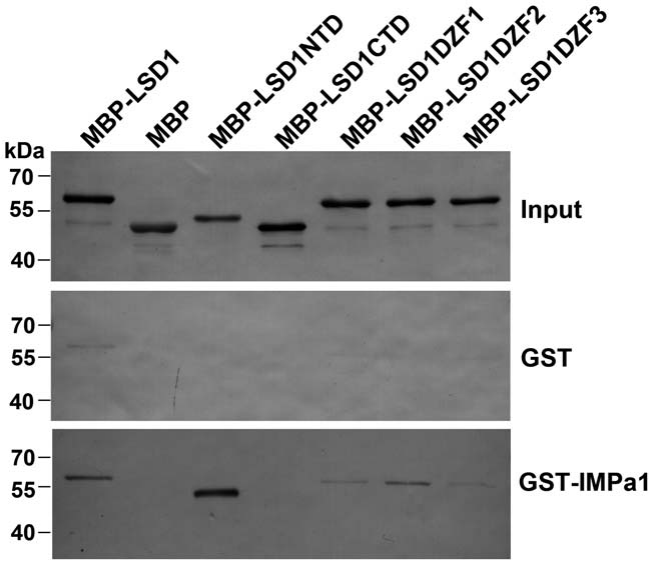
The NLS of PsLSD1 associates with AtIMPα1 *in vitro*. Purified MBP-PsLSD1, MBP, MBP-PsLSD1NTD, MBP-PsLSD1CTD, MBP-PsLSD1ΔZF1, MBP-PsLSD1ΔZF2, and MBP-PsLSD1ΔZF3 were incubated with GST-AtIMPα1 or GST (control) bound to MagneGST™ glutathione particles, respectively. Pulled-down proteins and “Input” sample (purified MBP fusion proteins) were detected by Western blot using an anti-MBP polyclonal antibody. The experiments were performed two times with similar results.

Next we carried out GST pull-down assay to analyze the interaction between the NLS of PsLSD1 and AtIMPα1. Figure 4 showed that the N-terminal domain, which served as the NLS, but not the C-terminal domain of PsLSD1 directly bound to AtIMPα1 *in vitro*; the N-terminal domain of PsLSD1 bound to AtIMPα1 with a greater intensity than full length PsLSD1 did (Figure 4). Moreover, deletion of the first, second, or third zinc finger significantly decreased the binding of PsLSD1 to At IMPα1 (Figure 4), suggesting that the zinc fingers are involved in direct interaction with importin α. Thus, our data suggest that the NLS of PsLSD1 directly binds to importin α *in vitro*.

## Discussion

### PsLSD1 is localized in the nucleus

Consistent with AtLSD1 [1], [2], [8], our data indicate that PsLSD1 contains three LSD1-type zinc finger motifs (Figure S1), and negatively regulates plant PCD (Figure 1C and 1D), demonstrating that PsLSD1 is a homolog of AtLSD1. Subcellular localization analysis of GFP-PsLSD1 indicates that PsLSD1 is localized in the nucleus (Figure 2B). This result was further supported by subcellular localization analysis of PsLSD1 deletion and point mutants (Figure 2C, 2D, 2E and 2F), and the finding that PsLSD1 interacts with nuclear import receptor importin α (Figure 3A, 3B, and 3C). Taken together, our data demonstrate that LSD1 is localized in the nucleus.

Consistent with our finding, LSD1 has been hypothesized to act as a transcription factor, since its C-terminal domain shows relatively low homology to several known DNA-binding and transcription factors [8]. In addition, LSD1 has been shown to regulate SA-induction of copper zinc superoxide dismutase [3] and the expression of *CAT1* [5]. Thus, the nuclear localization of PsLSD1 further suggests that LSD1 may function as a transcription regulator, either a transcription factor or a nuclear protein modulating the activity of transcription factors.

### The LSD1-type zinc finger motifs are a novel NLS

In this study, we provide several lines of evidence that the three zinc finger motifs of PsLSD1 are a functional NLS. First, the three zinc finger motifs are necessary for PsLSD1 to localize in the nucleus (Figure 2C, bottom panel; Figure 2D). Second, the three zinc finger motifs are sufficient for PsLSD1 to localize in the nucleus (Figure 2C, upper panel). Third, tertiary structure and basic residues of each zinc finger motif are required for PsLSD1 to localize in the nucleus (Figure 2E and 2F). Lastly, the three zinc finger motifs can be recognized by classical NLS receptor importin α (Figure 3 and 4). Thus, our data demonstrate that the three zinc finger motifs of PsLSD1 can function as an NLS.

Consistent with our findings, zinc finger motifs have been identified as the NLS of several zinc finger proteins [15], [22], [23], [24], [25], [26], [27], [28], [29], [30], [31]. Moreover, like NGFI-A [15], EKLF/KLF1 [27], [28], and Sp1 [24], our data indicate that all the three zinc finger motifs are required for PsLSD1 nuclear localization (Figure 2D). However, it is worthwhile to note that zinc finger motifs of these proteins are C_2_H_2_-type, whereas all the three zinc finger motifs of PsLSD1 are LSD1-type. There are several differences between these two types of zinc finger motifs. First, the C_2_H_2_-type zinc finger motifs (InterPro accession number: IPR007087) extensively exist in all sorts of organisms, whereas LSD1-type zinc finger motifs (InterPro accession number: IPR005735) mainly exist in plants (http://www.ebi.ac.uk/interpro/). Second, the C_2_H_2_-type zinc finger motifs contain two conserved cysteine and two conserved histidine residues [32], whereas the LSD1-type zinc finger motifs are characterized by CxxCRxxLMYxxGASxVxCxxC [8]. Third, the secondary structure of C_2_H_2_-type zinc finger motif consists of two β sheets and one α helix [32], whereas PSIPRED program analysis showed that the secondary structure of LSD1-type zinc finger motif is composed of three β sheets (Figure S2). Lastly, most of the C_2_H_2_-type zinc finger motifs acting as an NLS are involved in the binding of DNA or RNA [15], [22], [23], [24], [27], [28], [29], [30], whereas LSD1-type zinc finger motifs are implicated in the interaction with other proteins, including bZIP10 [9], MC1 [10] and GILP [11]. Thus, the three LSD1-type zinc finger motifs of PsLSD1, which are totally different from the C_2_H_2_-type zinc finger motifs, are a novel NLS.

### The NLS of PsLSD1 interacts with importin α

In the classical nuclear import pathway, importin α is involved in the recognition of classical NLSs, and the import of nuclear proteins into the nucleus with the assistance of importin β [12]. It has been reported that C_2_H_2_-type zinc finger motifs of EKLF and ZIC3 serve as an NLS and directly interact with importin α [28], [31]. Interestingly, our data also indicate that the LSD1-type zinc finger motifs of PsLSD1 directly bind to the classical NLS receptor importin α *in vitro* (Figure 3 and 4). This implies that LSD1 may be transported into the nucleus through its interaction with importin α, and suggests that LSD1-type zinc finger motifs may be recognized as a novel type of classical NLS.

It has been reported that tertiary structure of C_2_H_2_-type zinc finger motifs is necessary for nuclear localization of NGFI-A and Sp1 [15], [33]. Moreover, basic amino acids within C_2_H_2_-type zinc finger motifs are essential for nuclear localization of EKLF/KLF1 and ZIC3 [27], [31]. Consistent with these findings, our data indicate that both tertiary structure and basic amino acids of LSD1-type zinc finger motifs are essential for PsLSD1 nuclear localization (Figure 2E and 2F). Moreover, it has been reported that basic amino acids within the two zinc finger motifs cluster together in two regions on the ZIC3 protein surface, functioning as a bipartite-like NLS [31]. Taken together, we postulate that basic residues in the three LSD1-type zinc finger motifs may be brought into close proximity within the three-dimensional structure of LSD1, forming an NLS that can be recognized by importin α.

**Figure 5:**
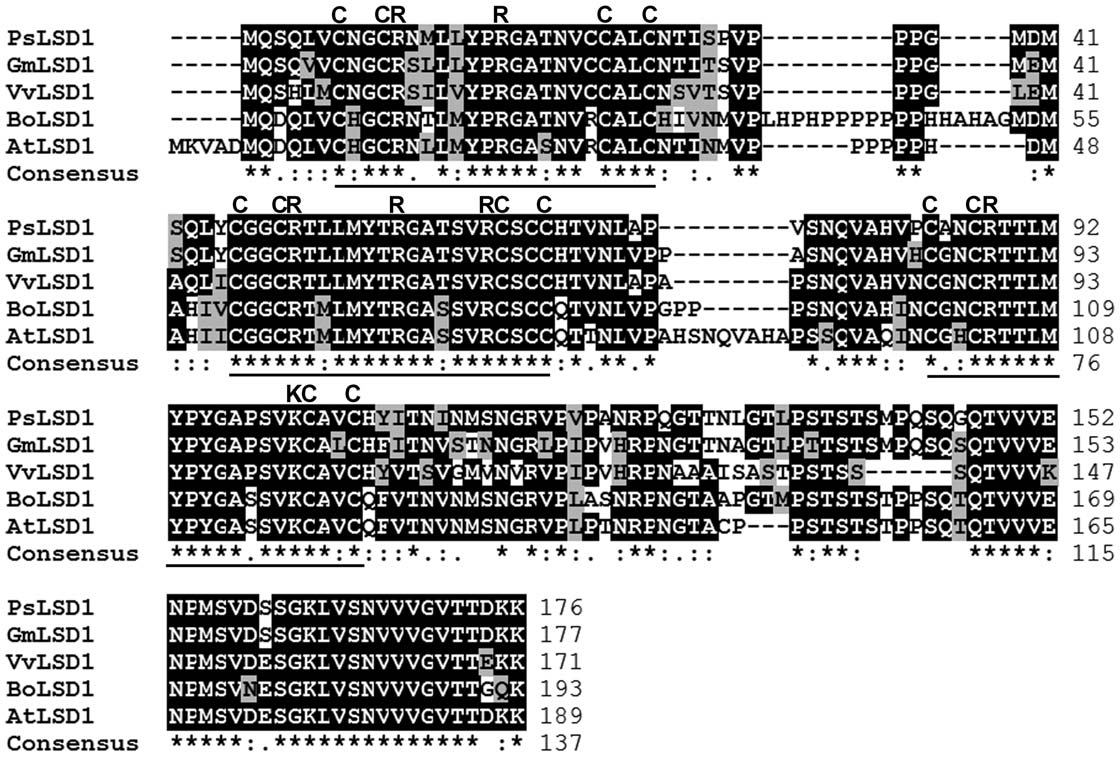
The three LSD1-type zinc finger motifs of LSD1s are highly conserved. The LSD1s are shown in the following order: *Pisum sativa* (*Ps*) LSD1 (GenBank accession number: AAY40471.1), *Glycine max* (*Gm*) LSD1 (UniProtKB accession number: C6TGF7), *Vitis vinifera* (*Vv*) LSD1 (UniProtKB accession number: A7QC17), *Brassica oleracea* (*Bo*) LSD1 (UniProtKB accession number: Q8W195), and *Arabidopsis thaliana* (*At*) LSD1 (UniProtKB accession number: P94077). Amino acid sequences of LSD1s were analyzed by the ClustalW2 program (http://www.ebi.ac.uk/Tools/clustalw2/index.html). The three conserved zinc finger motifs are underlined. The zinc chelating cysteine residues and conserved basic residues within the zinc finger motifs are shown in the upper region of the sequences.

As discussed above, the three LSD1-type zinc finger motifs and basic residues within these motifs are essential for PsLSD1 nuclear localization. Sequence alignment analysis indicates that both these motifs and basic residues are highly conserved in LSD1s (Figure 5). Thus, our findings that the three zinc finger motifs of PsLSD1 function as a NLS and interact with importin α may apply to all the LSD1s.

In summary, our results suggest that the three LSD1-type zinc finger motifs of LSD1 are a novel NLS and can be recognized by importin α, and may shed light on the nuclear localization mechanism of LSD1. Furthermore, the nuclear localization of PsLDS1 suggests the possible function of LSD1 as a transcription regulator involved in regulating PCD. The molecular mechanism of LSD1 regulating PCD should be investigated in the future.

## Materials and Methods

### Plant growth

*Arabidopsis* seeds were soaked in 70% ethanol plus 0.01% Triton X-100 for 5 min, washed five times with sterile water, and then sown on MS medium [4.3 g/L MS salts (Sigma, USA), 3% sucrose, 0.8% agar, pH5.7]. After stratification at 4°C for 2–4 days, plates were transferred to a tissue culture room at 22°C under 9 h photoperiod. After one week growth, seedlings were potted in soil and grown in a growth chamber at 22°C and 60% relative humidity under 9 h photoperiod (for SA spray and protoplast preparation) or in the tissue culture room at 22°C and 50–70% relative humidity under 16 h photoperiod (for construction of transgenic plant).

### Construction of transgenic plant in the *lsd1–2* background

The *lsd1-2* (SALK_042687) seeds [34] were obtained from the *Arabidopsis* Biological Resource Center (Ohio State University, Columbus). The homozygous mutant was identified by PCR using the T-DNA-specific primer and *AtLSD1*-specific primers. RT-PCR analysis was used to confirm the absence of *AtLSD1* mRNA in the homozygous mutant *lsd1-2*.

To generate the construct for over-expression in *Arabidopsis*, *PsLSD1* cDNA was amplified and cloned into our previously constructed vector pRTL-HA [11] via *Kpn*I/*Bam*HI to generate pRTL-HA-PsLSD1. The expression cassette *35S:HA-PsLSD1* was cleaved from pRTL-HA-PsLSD1 via *Pst*I and cloned into the *Pst*I site of pCAMBIA1381 to generate 1381-HA-PsLSD1.

The *Agrobacterium* haboring the construct 1381-HA-PsLSD1 were used to transform the homozygous mutant *lsd1-2* by the floral-dip method [35]. Independent transformants were analyzed for the expression level of HA-PsLSD1 by Western blot using an anti-HA monoclonal antibody (Sigma, USA).

### Ion leakage measurement

Five-week-old plants were sprayed with 2 mM SA (Sigma, USA) until liquid dripped off the leaves and covered with a plastic film for 24 h [36]. After 3 days, four leaf discs (4.5 mm diameter) per sample were used for ion leakage measurement. Ion leakage measurement was performed essentially as previously described [11].

### Subcellular localization

To generate the constructs GFP-PsLSD1, GFP-PsLSD1NTD, GFP-PsLSD1CTD, and GFP-PsLSD1ΔZF1, the corresponding fragments of *PsLSD1* were amplified by specific primers and cloned into the GFP fusion expression vector pAVA121 [37] via *Bgl*II/*Xba*I.

To generate the fragments PsLSD1ΔZF2 and PsLSD1ΔZF3, the second and third zinc finger regions of *PsLSD1* were deleted by recombinant PCR, respectively. To generate the mutated fragments PsLSD1C1A, PsLSD1C2A, PsLSD1C3A, PsLSD1R1A, PsLSD1R2A, and PsLSD1R3A, the coding region of *PsLSD1* were mutated by recombinant PCR using specific primers with corresponding mutated nucleotides, respectively. All these fragments were cloned into pAVA121 via *Bgl*II/*Xba*I to generate GFP-fusion expression constructs.

*Arabidopsis* mesophyll protoplast preparation and transfection were performed according to the protocol published by Yoo et al [38]. After incubation at room temperature for 12–16 h, nucleic acids of transfected protoplasts were stained with 4,6-diamidino-2-phenylindole (DAPI, 1 µg/mL; Sigma, USA) at room temperature for 30 min. After staining, the samples were visualized under a fluorescent microscope (Leica, Germany).

### Yeast two-hybrid assay

To generate the construct pGBK-AtIMPα1, pGBK-AtIMPα3, and pGBK-AtIMPα4, the coding regions of AtIMPα1, AtIMPα3, and AtIMPα4 were cleaved from the constructs GST-AtIMPα1, GST-AtIMPα3, and GST-AtIMPα4 [21] via *Eco*RI/*Bam*HI and cloned into the bait vector pGBKT7 (Clontech, USA), respectively. To generate the construct pGBK-AtIMPα2, the coding region of AtIMPα2 was cleaved from the construct GST-AtIMPα2 [21] via *Eco*RI/*Sal*I and cloned into the bait vector pGBKT7 (Clontech, USA). To generate the construct pGAD-PsLSD1, the coding region of PsLSD1 was amplified and cloned via *Eco*RI/*Sal*I into the prey vector pGADT7-Rec (Clontech, USA). pGAD-PsLSD1 was co-transformed with pGBK-AtIMPα1, pGBK-AtIMPα2, pGBK-AtIMPα3, pGBK-AtIMPα4, and pGBKT7 into yeast AH109, respectively. The β-galactosidase activity of the resulting clones was measured. Yeast tansformation and β-galactosidase activity assay were performed as the manufacturer's protocols (Clontech, USA).

### *In vitro* pull-down assay

To generate the constructs MBP-PsLSD1, MBP-PsLSD1NTD, MBP-PsLSD1CTD, MBP-PsLSD1ΔZF1, MBP-PsLSD1ΔZF2, and MBP-PsLSD1ΔZF3, the various deletion fragments of *PsLSD1* were cleaved from the corresponding GFP fusion constructs via *Bgl*II/*Xba*I and cloned into the MBP-fusion expression vector pMAL-c2X [New England Biolabs (Beijing) LTD., China] via *Bam*HI/*Xba*I, respectively. The construct GST-AtIMPα1 was provided by Dr. Gelvin [21].

All constructs were transformed into *Escherichia coli* BL21 (DE3) cells. BL21 (DE3) cells were treated with isopropyl-β-D-thiogalactoside to induce fusion protein expression. For batch purification of MBP fusion proteins, BL21 bacteria were lyzed by sonication in column buffer (20 mM Tris-HCl pH 7.4, 200 mM NaCl, 1 mM EDTA, 1 mM phenylmethylsulphonyl fluoride, 4 µg/mL aprotinin, 4 µg/mL leupeptin), and then the lysates were bound to amylose resins [New England Biolabs (Beijing) LTD., China]. The protein-bound amylose resins were washed three times with column buffer, and then eluted with column buffer containing 10 mM maltose.

GST-AtIMPα1 or GST proteins were bound to MagneGST™ glutathione particles, and then i*n vitro* GST fusion pull-down assays were performed according to the manufacturer's protocols (Promega, USA) except that purified MBP fusion protein was used. Pulled-down proteins were detected by Western blot using an anti-MBP polyclonal antibody [New England Biolabs (Beijing) LTD., China].

### Bimolecular fluorescence complementation assay

The coding region of AtIMPa1 was amplified and cloned via *Sac*I/*Xba*I into the YN fusion expression vector YN-EE [39]. The coding region of PsLSD1 was amplified and cloned via *Sal*I/*Xba*I into the YC fusion expression vector YC-HA [39]. YC-PsLSD1 and YC-HA (negative control) were co-transfected with YN-AtIMPa1 into *Arabidopsis* protoplasts, respectively. After incubation at room temperature for 12–16 h, nucleic acids of transfected protoplasts were stained with DAPI and then the transfected protoplasts were visualized under a fluorescent microscope (Leica, Germany).

## Supporting Information

Figure S1 **PsLSD1 cDNA sequence and deduced amino acid sequence.** The three LSD1-type zinc finger motifs are underlined.
(TIF)

Figure S2 **Secondary structure analysis of PsLSD1.** Amino acid sequence of PsLSD1 was analyzed by the PSIPRED program (http://bioinf.cs.ucl.ac.uk/psipred/).
(TIF)
