# Supplementary figures and images for: The LSD1-Type Zinc Finger Motifs of Pisum sativa LSD1 Are a Novel Nuclear Localization Signal and Interact with Importin Alpha
Source: PLoS One. 2011 Jul 19;6(7):e22131. doi: 10.1371/journal.pone.0022131 (PMC3139611; doi:10.1371/journal.pone.0022131)

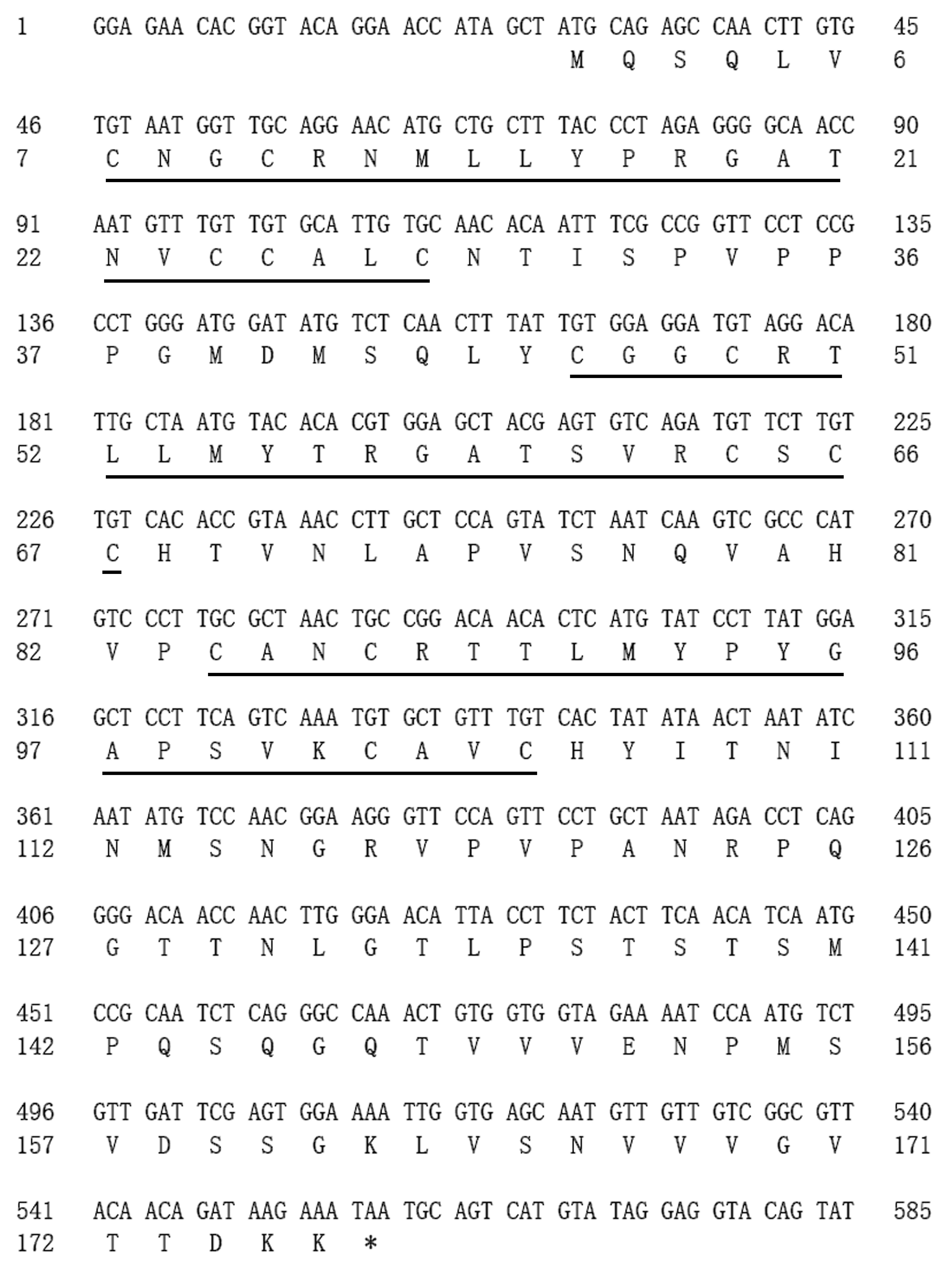

Supplement: Figure S1 — PsLSD1 cDNA sequence and deduced amino acid sequence. The three LSD1-type zinc finger motifs are underlined. (TIF) [file pone.0022131.s001.tif]

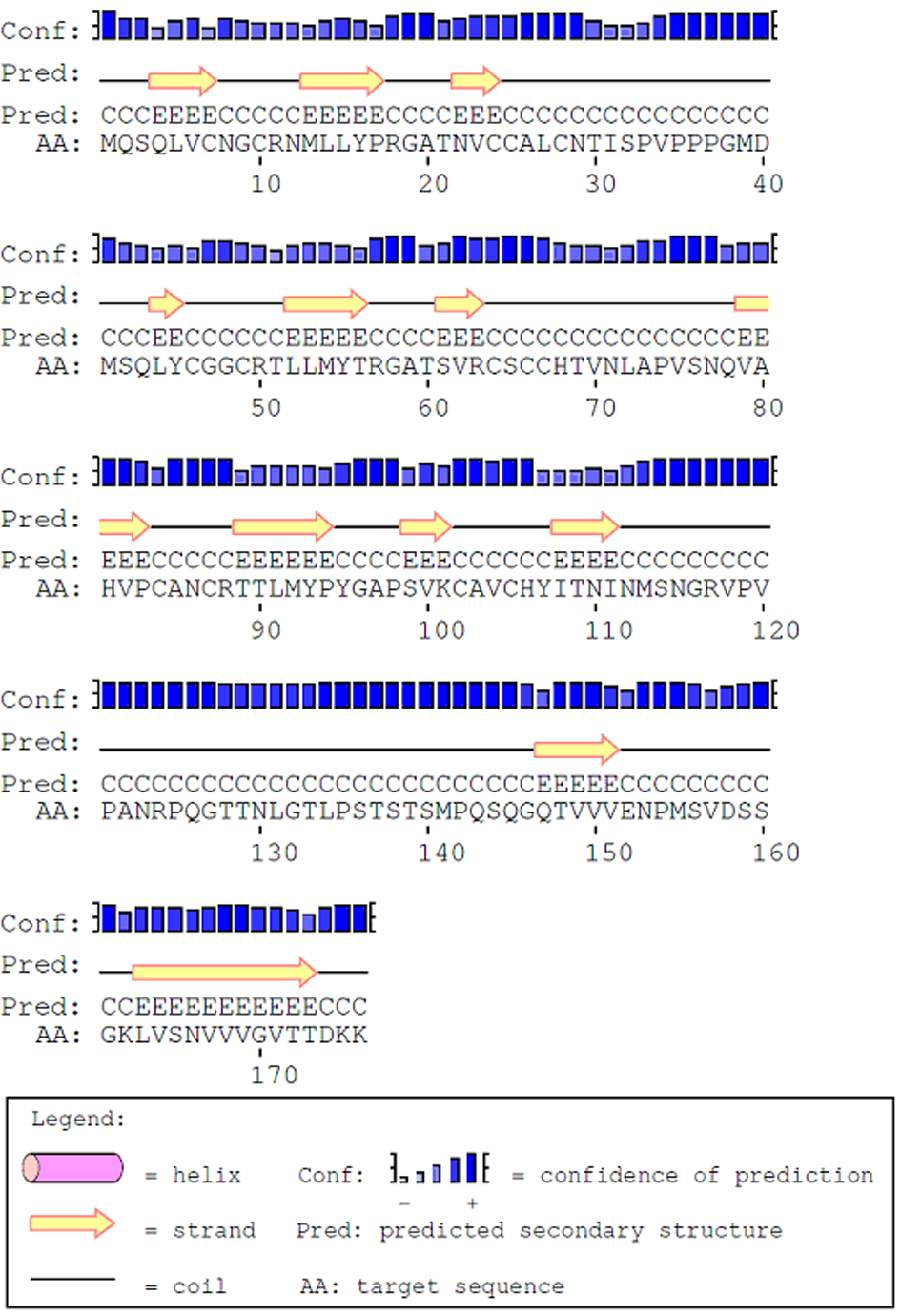

Supplement: Figure S2 — Secondary structure analysis of PsLSD1. Amino acid sequence of PsLSD1 was analyzed by the PSIPRED program (http://bioinf.cs.ucl.ac.uk/psipred/). (TIF) [file pone.0022131.s002.tif]
